# The eosinophilic variant of chronic myeloid leukemia

**DOI:** 10.17179/excli2021-4462

**Published:** 2021-11-26

**Authors:** Stephen E. Langabeer

**Affiliations:** 1 Cancer Molecular Diagnostics, St. James's Hospital, Dublin, Ireland

## ⁯⁯⁯

***Dear Editor,***

Chronic myeloid leukemia (CML) is a myeloproliferative neoplasm, characterized by the presence of the *BCR-ABL1* oncogene. The common presenting hematological feature is a leucocytosis due to neutrophils in various stages of maturation. Although an absolute eosinophilia and basophilia are common for patients presenting in chronic phase the median eosinophil count is only 2 % of the total white blood cell count (Hoffmann et al., 2015[5]). Rarely, CML may present hematologically as an isolated thrombocytosis, erythrocytosis, basophilia or eosinophilia: the eosinophilic variant of CML is extremely rare with only sporadic case reports documenting this type of presentation. Here, those patients with the eosinophilic variant of CML are collated in order to determine any salient features that may improve outcome.

A literature search was performed using the search terms “eosinophilic variant” or “eosinophilia” and “CML” to identify patients. Cases analyzed were restricted to those in English language journals and published in the tyrosine kinase inhibitor (TKI) era. Five individual cases CML deemed to have the eosinophilic variant of CML were identified (Aggrawal et al., 2009[1]; Chhabra and Verma, 2020[2]; Gotlib et al., 2013[3]; Ray et al., 2021[6]; Yerrapotu et al., 2020[7]) with the patient clinical details summarized in Table 1(Tab. 1) (References in Table 1: Aggrawal et al. 2009[1]; Chhabra and Verma, 2019[2]; Gotlib et al., 2003[3]; Ray et al. 2021[6]; Yerrapotu et al., 2020[7]).

Acknowledging possible publication bias due to reporting of cases with novel or atypical features, it can be seen that all cases are in male patients: one further case report (in Japanese) documents an 80 year-old female CML patient presenting with the eosinophilic variant and achieving a short lived complete cytogenetic remission with Imatinib (Haseyama et al., 2018[4]). Given the median age of CML diagnosis is 55 years (Hoffmann et al., 2015[5]), the median age of these patients (34 years) appears substantially younger though this may be an effect of the small cohort size. Splenomegaly is largely absent with patients often presenting with cutaneous manifestations, likely due to circulating pathological levels of eosinophils deregulating host defense mechanisms, immune responses and tissue damage pathways. Long term follow-up of imatinib therapy remains undocumented in this cohort with the potential treatment with alternative tyrosine kinase inhibitors not yet explored.

Reporting of further cases of this unusual variant of CML is wholly warranted in order to better understand its pathological nature and to determine optimal TKI therapy.

## Conflict of interest

The author declares that he has no conflict of interest.

## Figures and Tables

**Table 1:**
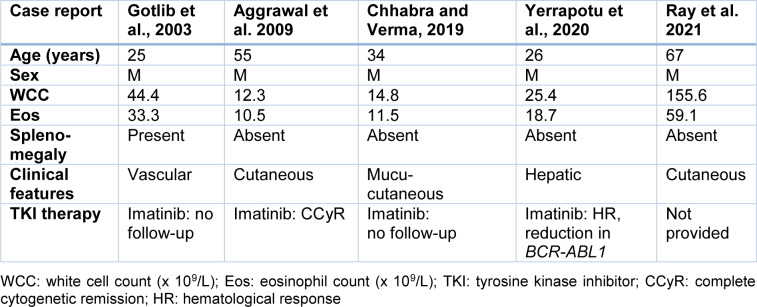
Presenting features of patients with the eosinophilic variant of chronic myeloid leukemia
